# Supplementary material for: Matrix WaveTM System for Mandibulo-Maxillary Fixation—Just Another Variation on the MMF Theme?—Part II: In Context to Self-Made Hybrid Erich Arch Bars and Commercial Hybrid MMF Systems—Literature Review and Analysis of Design Features
Source: Craniomaxillofac Trauma Reconstr. 2025 Jul 15;18(3):33. doi: 10.3390/cmtr18030033 (PMC12286215; doi:10.3390/cmtr18030033)
Supplement: Supplementary file 1 [file cmtr-18-00033-s001.zip › cmtr-3499289 -Part II Supplemental Electronic Content_edcpc_grat.pdf]

Supplemental Electronic Content

for

Cornelius CP, Liokatis PG, Doerr T, Damir M, Fusetti S, Rasse M, Gellrich NC, Heiland M, Schubert W, Buchbinder D.
Matrix Wave™ System for mandibulo-maxillary fixation - just another variation on the Mandibulo-Maxillary Fixation theme? –
Part II: In context to selfmade hybrid Erich arch bars and commercial hybrid MMF systems –review of the literature and analysis of design features.
Craniomaxillofac Trauma Reconstr. 2025, *18*, 33

• *Table S1*

• Self-made / Modified Hybrid Erich Arch Bars – Summary of Clinical Studies

Table S 1

	Characteristics / MMF Features								Timing	Intraoperative Complications					Postoperative Complications						Overall Outcome
Authors	Fracture Types – Treated	Hybrid Arch Bar Design – Test Group	Study Type	Compared MMF Modality Control	Sample Size/ Patient Number	Spanning Width of Hybrid Bars	Number of screws for Hybrid Bar Fixation	Number of Circum-dental Wires / Hybrid MMF Screws	Appli-cation Time	Pain	Glove Tears	Skin Puncture	Tooth Root Damage Imaging Con-firmation	MMF Duration / Devices in Place	Occlusion	Stability	Mucosal Over-growth / Screw Coverage	Loose Screws	Device Touch Ups Required	Oral Hygiene (Indices)	
**Hassan et al 2018**	favorable and unfavorable fractures of the mandible	presum-ably accor-ding to De Queiroz; see Fig. 1 A	not spec-ified, pre-sum-ably pro-spective	Conven-tional EABs & MMF Screws	Hybrid: n = 30 EAB: n = 30 MMF screws: n = 30	Full arch	4 x 1.5 screws per arch; in total 8 or more if needed after predrilling	ø	Hybrid: ø EAB: ø MMF Screws: ø	ø	ø	ø	ø	ø	ø	ø	ø	ø	ø	Hybrid (best) > EAB > MMF screws [OHI-S: hybrid < EAB < MMF screws	Hybrid Arch Bars = quicker, safer and more efffient method in comparison to EAB and MMF Screws
**Rothe et al 2018**	mandible fractures in symphysis, parasymphysis and condylar process	see Fig. 1 C	pro-spective	MMF screws	Hybrid: n = 10 MMF Screws: n = 10	Full arch, Splitting of bars in multifrag mentary fractures to ease reductio n	4 x 1.5 screws per arch; in total 8 or more if needed after predrilling	4 - 6 x 2.0 MMF Screws length 8 mm after predrillin g	Hybrid: 29 minutes; MMF Screws: 16 minutes	ø	ø	ø	Hybrid: 1 case; MMF Screws: 1 case	≤ 45 days	ø	Hybrid > MMF Screws	MMF Screws coverage: partial of all screws post 15 days, full of 4 screws post 45 days; Hybrid Screws: full of 1 screw	MMF Screws: 1 case	ø	[TGP-I] Hybrid: high level - no change during follow up. MMF Screws: from initially 2.1 ± 0.5 to	MMF screws are the quicker and easier method with better maintenance of oral hygiene, Hybrid Arch Bars are more stable
**Rothe et al 2019**	mandible fractures in symphysis, parasymphysis and condyle	see Fig. 1 C	rando-mized clinical trial	Conven-tional EABs & MMF Screws	Hybrid: n = 10 EAB: n = 10 MMF Screws: n = 10		4 x 1.5 screws per arch; in total 8 or more if needed after predrilling	EAB wire: ø ; 4 - 6 x 2.0 MMF Screws length 8 mm after predrillin g	Hybrid: 29 minutes; MMF Screws 16 minutes; EAB: 110	ø	Hybrid: none; MMF Screws : none; EAB: 6 cases	ø	Hybrid: 1 case; MMF Screws: 1 case	≤ 45 days	ø	EAB > Hybrid > MMF Screws	identical with previous study	MMF Screws: 1 case	ø	[TGP-I] Hybrid & MMF Screws : see study 2018; EAB from 1.5±0.4 to 2.3±0.3	MMF screws are quickest and easiest method followed by Hybrid arch bars, both with low risk of wire stick punctures, Oral hygiene: MMF Screws > Hybrid > EAB, Stability: EAB > Hybrid > MMF
**Pathak et al 2019**	favorable and unfavorable fractures of the mandible; maxillary fractures affecting occlusion	see Fig. 1 C	pro-spective	Conven-tional EABs	Hybrid: n = 10 EAB: n = 10	Full Arch	4 x 1.5 screws per arch; in total 8 or more if needed after predrilling	ø	Hybrid: 27.2 ± 3.53 minutes; EAB: 82.5 ± 18.9 minutes	Discomfort during Place-ment / Removal Hybrid: 30 %/ 20 %; EAB: 100 %/ 100 %	Hybrid: 0%; EAB: 30 %	ø	Hybrid: 1 case	≥ 6 weeks	ø	Adequatel y stable Hybrid: 90 % (n=9/10 patients) EAB: 80 % (n=8/10 patients)	Hybrid: occurrence in n = 4/10 patients	ø	ø	Hybrid > EAB	Hybrid Arch Bars – Downside: tooth root damage. Advantages: easy to place, excellent intraOP fixation. Post OP – atraumatic to soft tissues, improved oral hygiene, high patient compliance – Good alternative to EABs
**Venugo-palan et al 2020**	Mandibular Fractures including Condylar Process Fracture Le Fort Fractures	see Fig.1 D	pro-spective	Conven-tional EABs	Hybrid: n = 16 EAB: n = 16	ø	4 x 1.5 screws (2 anteriorly and 2 posteriorly)	ø	Hybrid: 21.8 ± 1.3 minutes; EAB: 76.3±6.2 minutes	Visual Analo-gue Scale: Hybrid: 15.9; EAB:49.2	ø	Hybrid: 18.8%. EAB: 53.6%	Increased non-vital tooth responses in EABs	4 - 6 weeks Elastics	Satisfactor y in both groups; 1 unstable case per group	Instability - Hybrid: 1 case; EAB: 21 cases	ø	Hybrid: 1 screw in n = 4 /16 cases ;	Hybrid: 87.5 % (n=14/16); EAB: 93.8 % (n=15/16)	Hybrid better than EAB- [OHI-S: Hybird B < EAB]	Hybrid Arch Bars more efficient than EABs – diminishing operative time, skin punctures and periodontal trauma

Table 1 S1. Self-made / Modified Hybrid Erich Arch Bars – Summary of Clinical Studies. Table 1 Legend: Patient age ≥ 18 years in all studies; Ø = not specified / no information; Hybrid = Modified Erich Arch Bar (EAB); [OHI-S] = Oral Hygiene Index-Simplified (Greene and Vermillion 1964)[12]; [TGP-I] = Turesky-Gilmore (Glickman) Plaque-Index (1970)[13] (Modification of Quigley-Hein. Index - 1962)[14];

[**Table S1** is attached as an Excel File: Table S1 Self-made Hybrid Arch Bars.xlsx]

References – Table S1(eContent):

104. Greene, J.C.; Vermillion, J.R. The simplified oral hygiene index. *J. Am. Dent. Assoc.* **1964**, *68*, 7–13. https://doi.org/10.14219/jada.archive.1964.0034.

105. Turesky, S.; Gilmore, N.D.; Glickman, I. Reduced plaque formation by the chloromethyl analogue of victamine C. *J. Periodontol.* **1970**, *41*, 41–43. https://doi.org/10.1902/jop.1970.41.41.41.

106. Quigley, G.A.; Hein, J.W. Comparative cleansing efficiency of manual and power brushing. *J. Am. Dent. Assoc.* **1962**, *65*, 26–29. https://doi.org/10.14219/jada.archive.1962.0184.

• *Text S1*

• Summaries of Self-made / Modified Hybrid Erich Arch bars – Clinical Studies

Summaries of each study listed in Table S1:

None of these six studies adopts all the assessment parameters disseminated in the respective literature. Therefore some parameters –- are not displayed in Table 1. The spanning width of EABs as well as the total OR time is not specified in any study. Concomitant ORIF therapy was performed in 2 studies: Hassan et al. (2018)[9] did not detail on the type of osteosynthesis, while Venugopalan et al. (2020)[10] utilized miniplates across both comparative groups – Hybrid versus EAB.

Only two studies used tooth vitality to detect iatrogenic tooth root damage instead of imaging, Venugopalan et al. (2020)[10] and Elhadidi et al. (2023)[11] used electronic pulp testing. Surprisingly, Venugopalan et al. (2020)[10] observed significantly increased nonvital tooth responses in the EAB compared to the Hybrid patient group.

Elhadidi et al. (2023)[11] – probably not expecting tooth injuries in tooth-borne EABs – performed vitality tests preoperatively and at removal in the Hybrid group only. Eight teeth (central and lateral incisors, second premolars and first molars) in close proximity to screws were tested with no difference at the two testing times.
The study of Venugopalan et al. (2020)[10] is also the only one to report on periodontal trauma / mucosal tears, which was found in 4 patients of the EAB group.
The two studies by Rothe et al. (2018)[6] and (2019)[7] are identical down to the results and wording. The only difference is that the later study adds data for conventional EABs. Of particular note are photographs showcasing hybrid arch bars with ill-advised placement in the mobile mucosa inducing inflammatory screw overgrowth in some studies.

In part, the results of the six studies are inconsistent and even contradictory (e.g. Hassan et al. 2018[9] versus Rothe et al. 2019)[7]. One potential explanation for this are small patient sample sizes.
Quality of Life (QoL) issues in treatment with self-made hybrid arch bars were exclusively addressed by Pathak et al. (2019)[8] with QoL better in Hybrid than EAB patients.

References – Text S1(eContent):

6. Rothe, T.M.; Kumar, P.; Shah, N.; Shah, R.; Kumar, A.; Das, D. Evaluation of efficacy of intermaxillary fixation screws versus modified arch bar for intermaxillary fixation. *Natl. J. Maxillofac. Surg.* **2018**, *9*, 134–139. https://doi.org/10.4103/njms.NJMS_16_18.
7. Rothe, T.M.; Kumar, P.; Shah, N.; Shah, R.; Mahajan, A.; Kumar, A. Comparative evaluation of efficacy of conventional arch bar, intermaxillary fixation screws, and modified arch bar for intermaxillary fixation. *J. Maxillofac. Oral Surg.* **2019**, *18*, 412–418. https://doi.org/10.1007/s12663-018-1110-7
8. Pathak, P.; Thomas, S.; Bhargava, D.; Beena, S. A prospective comparative clinical study on modified screw retained arch bar (SRAB) and conventional Erich's arch bar (CEAB). *Oral Maxillofac. Surg.* **2019**, *23*, 285–289. https://doi.org/10.1007/s10006-019-00766-1.

9. Hassan, S.; Farooq, S.; Kapoor, M.; Shah, A. Comparative evaluation of modified Erich's arch bar, conventional Erich's arch bar and intermaxillary fixation screws in maxillo-mandibular fixation: A prospective clinical study. *Int. J. Med. Res. Res. Prof.* **2018**, *4*, 41–45. https://doi.org/10.21276/ijmrp.2018.4.4.011.
10. Venugopalan, V.; Satheesh, G.; Balatandayoudham, A.; Duraimurugan, S.; Balaji, T.S. A comparative randomized prospective clinical study on modified Erich arch bar with conventional Erich arch bar for maxillomandibular fixation. *Ann. Maxillofac. Surg.* **2020**, *10*, 287–291. https://doi.org/10.4103/ams.ams_20_20.
11. Elhadidi, M.H.; Awad, S.; Elsheikh, H.A.; Tawfik, M.A. Comparison of clinical efficacy of screw-retained arch bar vs conventional Erich's arch bar in maxillomandibular fixation: A randomized clinical trial. *J. Contemp. Dent. Pract.* **2023**, *24*, 928–935. https://doi.org/10.5005/jp-journals-10024-3613.

- ***Table S2***

- **SMARTLock Hybrid MMF System - Summary of Clinical Studies**

Table S2 – 1/3

Authors	Fracture Types / Indications	Treatment	Sample Size/ Patient Number	Number of screws for Hybrid Fixation	Number of Circumdental Wires / MMF Screws	Application Time	Pain	Glove Tears / Skin Puncture	Tooth Root Damage Imaging Confirmation	Occlusion	Stability	Mucosal Overgrowth / Screw Coverage	Loose Screws / Loose Circumdental Wiring	Device Touch Ups Required	Oral Hygiene (Indices)	Hard. ware Removal Time	Overall Outcome SMARTLock versus EAB or / and MMF screws
**Pilot Study**																	
**Nizam and Ziccardi 2014**	Mandible Fractures, Cysts, Ameloblastoma	ORIF, Protection against risk of pathologic fractures	SMARTLock Hybrid MMF: n = 10 patients	average of 12 Locking screws per application	ø	21.4 ± 6.4 minutes	none	none	radiographic root perforation in 2,5 % of screws	ø not specified	ø not specified	"gingival hyperpalsaia" n= 6 / 10 patients - limited to mandble	0.6 %	ø not specified	ø not specified	without Local Anesthesia	SMARTlock appears to provide adequate outcomes compared to other current MMF techniques– long term stability similar EABs, speed of application similar to MMF screws. reduced risk of wire stick injuries, adverse events: tooth root injuries
**Early Report**																	
**Kendrick et al 2016**	Single or Multiple Mandibular Fractures, Le Fort Fractures	Closed Reduction and ORIF	SMARTLock Hybrid MMF: n = 21 patients	7 Locking screws per arch	ø	14.4 minutes (range 9 - 24.7 minutes)	ø not specified n = 1 patient with MMF intolerance	none	n =24 / 319 teeth involvement of dentin n = 21, pulp n = 2, root fracture n = 1, treatment necessity n = 1	Malocclusion n = 3 patients (13 %)	ø not specified	n= 9 patients (38%)	Lost Screws n = 1	ø not specified	ø not specified	10.5 minutes (range 4.6 - 17 minutes)	SMARTLock Hybrid system is regarded an alternative to traditional EABs. Device appears to be safe,easy to use, and applied quickly. Surgeon' expertise required for appropriate case selection; system not ideal in all situations.
**Comparative Studies: SMARTLock – Hybrid MMF Devices - versus – Conventional EABs**																	
**Chao and Hulsen 2015**	Single or Multiple Mandibular Fractures	Closed Reduction or ORIF	SMARTLock Hybrid MMF: n = 25 patients; EABs: n = 21 patients	5 Locking screws per arch	n = 4 - 5 wires around premolars and molars / per arch	Hybrid: 42 minutes; EAB: 62 minutes	Hybrid: n = 1 ; EAB: n = 1	Hybrid and EAB: none	Hybrid and EAB: none	Malocclusion – Hybrid: none; EAB: n = 2	ø not specified	no mucosal necrosis; Hybrid – delayed Wound Healing n = 3	Hybrid and EAB: no hardware failures	ø not specified	ø not specified	Hybrid : 10 minutes; EAB: 8 minutes	Screw fixated Titanium arch bars are suggested as comparable alternative to conventional EABs. Bone-supported bars associated with shorter application time, otherwise similar to EAB
**Rani et al 2018 [Stainless Steel SMARTLock Facsimiles]**	Favorable and Unfovarable Maxillofacial Fractures	Probably: Closed Reduction, not specified otherwise	Hybrid: n = 20 patients; EAB: n = 20 patients	5 conventional (self-tapping) screws per arch	ø not specified	Hybrid: 34.9 ± 10 min; EAB: 53.1 ± 5.7 min	ø not specified	Hybrid: none EAB: 12	Hybrid: Dental damage 5 EAB: none	ø not specified	Hybrid: stable n = 19 EAB: stable n = 20	Hybrid: n = 7 EAB: none	ø not specified	ø not specified	Hybrid initially better EAB; EAB finally better Hybrid	ø not specified	Used hybrid MMF device (i.e stainless steal SMARTLock Replica) may be a comparable MMF alternative to EABs secured with circumdental wires
**Bouloux 2018**	One or More Mandibular Fractures	Closed Reduction or ORIF	SMARTLock Hybrid: n = 26 patients; EAB: n = 24 patients	ø not specified	ø not specified	Hybrid: 14.1 ± 8.4 minutes; EAB: 37.3 ± 15.1 minutes	ø not specified	ø not specified	n = 5	ø not specified Hybrid: Malunion n = 1, Fibrous Union n = 1, Nonunion n = 1; EAB: Malunion n = 1	ø not specified	ø not specified	ø not specified	ø not specified	ø not specified	ø not specified	No difference in length of total surgery time between Hybrid and EAB - 107.8 ± 73.3 minutes ± vs 117.2 ± minutes. Hybrids reduce the time for placement but do not appear to result in a reduction in the length of overall surgery treaing isolated mandibular fractures
**King and Christensen 2019**	Mandible Fracture requiring MMF	ø not specified	SMARTLock Hybrid: n = 47 patients; EAB: n = 43 patients	Small Arch Bar Type ; 5 Locking screws per arch	20 wires in total for both arches	Hybrid: 6.9 ± 3.1 minutes – [Use of Motor Screw Driver] ; EAB: 31.3 ± 9.3 minutes	ø not specified	Hybrid: 0.11 ± 0.32 per surgery EAB: 0.56 ± 0.91 per surgery	Hybrid and EAB: none	ø not specified	ø not specified	Gingival Appearance – Hybrid: Score 2.4 ± 1.0; EAB: 2.2 ± 0.8	Hybrid: 9.4 ± 17.7 %; EAB: 7.5 ± 10.6 % 1 circumdental wire swallowed	ø not specified	ø not specified	Hybrid: 10.5 ± 5.1 minutes; EAB: 17.9 ±10.7 minutes	Hybrid arch bars result in time savings for MMF placement and removal were associated with fewer glove perforations and hand injuries than EABs, i.e. greater margin of operator safety

Table S2 – continued 2/3

**Khelemsky et al 2019**	Non-condylar Mandibular Fractures	ORIF via transoral approach	Hybrid: n = 59 patients; EAB: n = 43 patients	10 Locking Screws per case	ø not specified	ø not specified instead total surgical time – Hybrid: 136 ± 2.7 versus EAB: 186.7 ±70.7 minutes	ø not specified	ø not specified	ø not specified	ø not specified	ø not specified	ø not specified	ø not specified	ø not specified	ø not specified	ø not specified	A significant amount of total surgical time was saved by Hybrids for unilateral (37.2 ±13.2 minutes) and bilateral fractures (55.8 ± 18.9 minutes); Results support the hypothesis that Hybrids are time savers in ORIF of mandible fractures. Hybrids save more time in bilateral fractures despite longer overall operative times
**Hamid and Bede 2021**	One or More Mandibular Fractures	Closed Reduction	Hybrid: n = 8 patients; EAB: n = 10 patients	8 Locking Screws per case	ø not specified	Hybrid: 41.6 ± 6 minutes; EAB: 61.6 ± 11.4 minutes	ø not specified	Hybrid: None; EAB: in n = 7 / 10 patients (70 %)	Hybrid and EAB: none	Hybrid and EAB: stable acceptable occlusion	Hybrid and EAB: No hardware failure	in 20 of a total of 64 screws (31,3 %)	Loosening 8 of 64 screws (12,5%)	ø not specified	Gingival Index Score (Loe/Silness) Hybrid: 2.12 ± 0.64; EAB: 2.6 ± 0.52	Hybrid: 14.2 ± 3 minutes [due to mucosal overgrowth]; EAB: 11.1 ± 2 minutes	Hybrids represent an alternative to EAB in mandibular fracture, requiring less application time and providing more safety for surgeons. Periodontal indices comparable between Hybrid and EAB during treatment period
**Sankar et al 2023 [Stainless Steel SMARTLock Facsimiles]**	Non-condylar Mandible Fractures	Closed Reduction	Hybrid: n = 21 patients; EAB: n = 23 patients	6 Conventional, non-self drilling screws per arch	ø not specified	Hybrid: 56.7 ± 17.9 minutes; EAB: 82 ± 12.2 minutes	ø not specified	Hybrid: None; EAB: Inner glove puncture 2; outer glove puncture 9; skin punture 1	2 lower incisor root injuries adjacent to 2 out 252 screws, asymptomatic after 2 weeks	ø not specified	Hybrid and EAB: comparable upper and lower arch bar stability through 4 weeks postOP	Hybrid: occured in all n = 21 patients; 137 of 252 screws heads covered 4 weeks postOP	Hybrid: loose screw n = 1; EAB: loose circumdental wires n = 2	ø not specified	OHI-S post OP: 1st week: Hybrid and EAB comparable; 2nd to 4th week: Hybrid better than EAB	ø not specified	Used hybrid MMF device (i.e stainless steal SMARTLock Replica) better than EAB: shorter application time of, reduced risk of skin prick injury, and improved oral hygiene
**Burman et al 2023. [Stainless Steel SMARTLock Facsimiles]**	Mandible and Midface Fractures	Closed Treatment	Hybrid n = 20; EAB n=21	up to 9 conventional (self-tapping) screws per arch	ø not specified	Hybrid: 23.3 ± 8,1 min; EAB: 86.4 ± 26.5 min	Hybrid: vesti-bular dis-comfort n=1 patient	ø not specified	Hybrid: None	Restoration of Preinjury Occlusion – Hybrid: 90 % patients; EAB: 81 % patients	Hybrid: n = 17 / 20 patients stable; EAB: n = 16/21 patients unstable	Hybrid: n = 17 / 20 patients (75 %)	Hybrid: n = 6 patients (30 %); EAB: n= 11 patients (52 %)	ø not specified	ø not specified	Hybrid: 30 minutes EABs: 19 minutes	Used hybrid MMF device (i.e stainless steal SMARTLock Replica) superior to EAB in terms of clinical convenience, application time, stability, prevention of wire injuries. Major concern: Mucosal overgrowth
**Comparative Studies: SMARTLock – Hybrid MMF Devices - versus – MMF Screws**																	
**Roeder et al 2019**	Mandibular Condylar Fractures – Isolated or plus Other Mandibular Fractures	Closed Reduction - ORIF of body fractures if appro-priate	SMARTLock Hybrid n = 7 patients; Synthes MMF Screws n= 5 patients	6 Locking Screws per case	4 Synthes MMF screws per case	Mean Total Operative Times - Hybrids only: 39,6 (range 31 - 54) minutes; MMF Screws only: 43 (range 38 - 48) minutes	ø not specified	ø not specified	ø not specified	Malocclusion Hybrid n = 1 (patient with panfacial fracture)	ø not specified	Hybrid: mucosal overgrowth more prevalent than in MMF Screws	ø not specified	ø not specified	ø not specified	ø not specified	Similar results in patient outcomes. Difference in average Operating Room Time is negligible (3–5 minutes) between Hybrid and MMF Screws – Hybrid plus ORIF Mean Total OR Time 81.3 Minutes vs MMF Screws plus ORIF 78.2 minutes. Great advantage of Hybrid is its flexibility and safety for surgeon. Advocated use of Hybrid system if MMF is required postoperatively to outweigh higher costs compared to MMF Screws.
**Aslam-Pervez et al 2020**	Mandibular Fractures	Closed Reduction (n= 24 patients), ORIF (n = 8 patients)	SMARTLock Hybrid: n = 19 ; Traditional MMF Screws n = 13	on average: 11 Locking screws	on average: 6 fixation screws	Hybrid: 25.92 minutes MMF Screws: 18.3 minutes	ø not specified	ø not specified	Hybrid: n = 1; MMF Screws: n = 2	ø not specified	ø not specified	Hybrid: n = 7; MMF Screws: n = 1 / Gingival Erythema Hybrid n = 8; MMF Screws n = 1	Hybrid: n = 1; MMF Screws: n = 1 Loose intermaxillary wire cerclages Hybrid : n = 1, MMF Screws: n = 4	ø not specified	ø not specified	ø not specified	Hybrids require more manipulation for ideal placement compared to individual MMF screws taking more application time. In contrast to MMF screws Hybrid devices associated with gingival overgrowth, but reduced incidence of screw loosening postOP, what indicates a clear advantage over MMF screws in terms of fracture stabilization and healing

Table S2 – Continued 3/3

**Comparative Studies: SMARTLock – Hybrid MMF Devices - versus – EABs and MMF Screws (4-Point Fixation)**																	
**Edmunds et al 2019**	Mandible Fractures	Closed Reduction	SMARTLock Hybrid: n = 15 patients; EAB: n = 27 patients ; 4-Point Fixation n = 51 patients	ø not specified	Number of circumdental Wires not specified ; 4 MMF Screws	Hybrid: 55.9 minutes (range 43.1 - 68.6); EAB: 98.7 minutes (range 89.2 - 108.2); 4-Point Fixation: 48.8 minutes (range 41.8 - 55.7)	ø not specified	ø not specified	Hybrid: None; EAB: Tooth damage n = 1 (4%); 4-Point Fixation: none	Malocclusion Hybrid: n = 2 (13 %); EAB: n = 3 (11 %); 4-Point Fixation: n = 5 (10 %)	ø not specified	ø not specified	Hybrid: n = 1; 4-Point Fixation: n = 1 Screw Loss 4-Point Fixation n = 1	ø not specified	ø not specified	Need to Return to OR for Hardware Removal. Hybrid: n = 1 (7%); EAB n = 17 (63 %); 4-Point Fixation n = 37 (72 %)	Bone-supported arch bars, i.e. SMARTLock hybrid MMF devices are an attractive alternative to both EABs and 4-point fixation. Complication outcomes are comparable. Operative time for placement is reduced in comparison to EABs and they have a lower likelihood of requiring removal in an operative setting.
**Systematic Reviews / Metaanalyses**																	
**Jain et al 2020**	Selection of 7 separate comparative studies on bone-supported arch bars (3 on hand-made hybrid EAB modifications plus 4 on SMARTLock hybrid) and EABs up to 2019. Outcomes of the 4 studies on SMARTLock hybrid MMF devices (Bouloux 2018, Rani et al 2018, King and Christensen 2019, Khelemsky et al 2019) are directly accessible in the listing above																Hybrid MMF Devices were considered the better option in comparison to EABs
**Sulistyani et al 2024**	Selection of 13 comparative studies on tooth-borne or bone-borne MMF devices, eligible for a final statistical summary. Bone-supported MMF devices not confined to hybrid MMF devices but including reports on MMF screws. Separate outcomes of 4 studies on SMARTLock devices (Bouloux 2018, Edmunds et al 2019, Hamid and Bede 2021, Sankar et al 2023) can be taken directly from the table of the article or the listing above.																Advantages and drawbacks are briefly itemized for tooth- and bone-borne MMF devices. Choice according to surgeon's preferences
**Kalluri et al 2024**	Selection of 24 studies on a variety of MMF types - MMF screws, Modified screw retained arch bars, commercial MMF devices (SMARTLock, OmniMax), Embrasure wires,DIMAC Wires, Leonard buttons, Vacuum formed splints, Hanger Plates, Eyelet wiring- in comparison with Erich Arch Bars as 'Gold Standard'. Unfortunate mix of the studies into inhomogenous categories and overlapping groupings leading to confusion somewhat misleading and flawing a meaningful interpretation of the results.																Attempt to formulate a comprehensive outcome opportune and affirming a positive trend for modern alternative MMF modalities

Table S2. SMARTLock Hybrid MMF System - Summary of Clinical Studies. Table S2 Legend: Patient age ≥ 18 years in all studies; Ø = not specified / no information; Hybrid = SMARTLock hybrid MMF System; [OHI-S] = Oral Hygiene Index-Simplified (Greene and Vermillion 1964)[12]; Three studies used stainless steel facsimiles / replicas of the titanium SMARTLock hybrid MMF System bone fixation with conventional

screws – Rani et al 2018[22], Sankar et al 2023[26], Burman et al 2023[27]. The table is sorted according to type (e.g. pilot study, comparative study, etc.) and not presented in a continuous chronological succession. [**Table S2** is attached as an Excel File: League_of_Commercial_Hybrid_MMF_Devices.xlsx].

References – Text S1(eContent):

104. Greene, J.C.; Vermillion, J.R. The simplified oral hygiene index. *J. Am. Dent. Assoc.* **1964**, *68*, 7–13. https://doi.org/10.14219/jada.archive.1964.0034. 22. Rani, E.B.; Reddy, S.; Amarnath, K.; Suresh Kumar, M.; Visalakhshi, G. Bone supported arch bar versus Erich arch bar for intermaxillary fixation: A comparative clinical study in maxillofacial fractures. *Int. J. Curr. Res.* **2018**, *10*, 69848–69850.

23. Sankar, H.; Rai, S.; Jolly, S.S.; Rattan, V. Comparison of efficacy and safety of hybrid arch bar with Erich arch bar in the management of mandibular fractures: A randomized clinical trial. *Craniomaxillofac. Trauma Reconstr.* **2023**, *16*, 94–101. https://doi.org/10.1177/19433875221080019.

24 Burman, S.; Rao, S.; Ankush, A.; Uppal, N. Comparison of hybrid arch bar versus conventional arch bar for temporary maxillomandibular fixation during treatment of jaw fractures: A prospective comparative study. *J. Korean Assoc. Oral Maxillofac. Surg.* **2023**, *49*, 332–338. https://doi.org/10.5125/jkaoms.2023.49.6.332.

• *Text S2*• Summaries of SMARTLock Hybrid MMF System – Clinical Studies

The clinical SMARTLock studies (Table S2) recorded the patient demographics, the indication and numerous assessment parameters. These included medical comorbidities, spanning width of the arch bars, number of retaining screws, screw insertion sites and vertical topographic arch bar placement, MMF device application time, device specific intraoperative complications (dental root damage, total operating room time, duration of intermaxillary fixation, oral hygiene, length of device retention, postoperative complications (screw / hardware loosening, mucosal overgrowth of screws, impairment of wound and/or bone healing), removal time, anesthetic method used during removal and overall estimation.

However these parameters did not have uniform definitions or grading scales nor were they consistently used, what poses serious limitations in comparing the studies.

The data of a 10 patient retrospective chart review from undergoing a variety of OMFS procedures using the SMARTLock Hybrid MMFTM System was presented in a pilot study on the system (Nizam and Ziccardi 2014)[15].

The indications included protection of extended mandibular bony defects against pathologic fractures, mandibular fractures and safeguarding after reconstruction of mandibular continuity defects.

The intent was to measure key parameters such as application time, dental root damage, stability of screw fixation, intra- and postoperative complications unique to the device and to implement assessment parameters for future clinical evaluations. Hence the paper attempted to formulate consistent and reproducible criteria for dental root perforations or screw loosening.

Root perforations by the authors criteria were defined, if pre- and postoperative imaging (Panoramic x-rays, CT scans) showed a greater than 50 % perforation of a screw into a root. Screw loosening was defined as a screw requiring early removal, if it was still locked in the arch bar but no longer in the alveolar bone.

The length of the devices (spanning width) in the mandible and maxilla was commonly adjusted to the full anterolateral dental arch from the rear of the first molars.
Patient follow up was a minimum of 3 weeks, extending for differing time periods depending on diagnosis and patient compliance.
12 ± 2.3 per patient screws were placed making a total of 120 in the whole series The average application time for the devices was 21.4 ± 6.4 minutes. Intermaxillary fixation was maintained for a mean of 3.4 ± 1.6 weeks. Removal of the devices occurred at 7.8 ± 3.6 weeks postoperatively under local anesthesia.
No wire-stick glove perforations or injuries to the operators were noted. Radiographically proven tooth root perforations were documented in 3 of 120 screws (2.5 %). Screw loosening was ascertained in 1 of 120 screws (0.8 %).
Gingival hyperplasia, granulation tissue due to a foreign body reaction, and mucosal overgrowth was confined to screws in the mandible. It was the most frequent postoperative complication and occurred in 6 of 10 patients.
In an early independent report by Kendrick et al. (2016)[17] 7 screws per arch were used to fasten the SMARTLock System, although the authors assumed that the number could be reduced to 5 screws and still ensure sufficient stability of the fixation.
The indications for treatment in the these 21 patients were predominantly single or less frequently multiple fractures in the mandible and a few Le Fort fractures. Before placing the SMARTLock devices, the fractures were manually reduced, if necessary. After establishing the preinjury occlusion and attaching intermaxillary wire cerclages to the connectors closed or open surgical techniques were used based on fracture classification and management plan.
The time required to set up the entire MMF assembly averaged to 14.4 minutes (range 9 – 24.7 minutes). No instances of wire-stick injuries were noted.
The intermaxillary wire fixation or heavy elastics were maintained for 12 to 50 days. The typical complications of surgical fracture repair included intraoral wound dehiscence, alocclusion, a loose osteosynthesis plate, a fatigue fracture of a superior border mini-plate at a mandibular angle fracture and a bony nonunion.
Specific complications attributable to the SMARTLock MMF System involved one patient with non-compliance for intermaxillary fixation, mucosal overgrowth of screws in nine cases (38 %), a lost screw, some loosened screws on removal, as well as mechanical irritation of the oral mucosa and lips.
The grade of tooth injuries caused by the application of a total of 319 bone screws was scrutinized postoperatively by 3-dimensional cone beam computer tomography (CBCT) imaging and revealed damaged dental root structures in 24 teeth. Dentin involvement occurred by far the most frequently (21 teeth). Perforations into the pulp chamber (1 tooth) or root fractures (2 teeth) were relatively rare events. The percentage of tooth root injuries per patient was not indicated, though. The time for removal of the hybrid MMF system was 10.5 minutes (range, 4.6 -17 minutes).
All the aforementioned findings paralleled the results of a previous retrospective cohort study, comparing the SMARTLock System to conventional EABs for treatment (closed or ORIF) of mandibular fractures in two groups. Each group consisted of 25 consecutive patients (Chao and Hulsen 2015)[16]. The installation time for the bone-borne device was considerably shorter than for the tooth-borne appliance (42 versus 62 minutes with ranges or standard deviations not reported). This was almost three times longer (42 versus 14.4 minutes) than noted by Kendrick et al. (2016)[17].
No glove perforations or wire-stick injuries were observed in the groups. Specific complications associated with bone-borne arch bars (screw complications and dental and mucosal injuries) were not recorded. The delayed wound healing of gingivobuccal incisions in the bone-borne group was attributed to the direct contact of the lugs and screws overlying the surgical access site. The postoperative complication rates were similar in both groups, those in the tooth-borne arch bar group due to occlusal discrepancies and prolonged pain rather than from wound dehiscence.
In succession of Chao and Hulsen (2015)[16] a prospective cohort study comparing bone supported arch bars to EABs secured with circumdental wires was published by Rani et al. {2018)[19]). Either of these MMF modalities was used for closed reduction of minimally displaced maxillofacial fractures. The study

comprised 40 patients divided equally. The bone supported arch bars were made of stainless steel and looked identical to the
embodiment of the SMARTLock Hybrid MMF System (facsimile/replica). These arch bars were fastened with 2.0 screws, five 6 mm long screws in the mandible and five 8 mm in the upper jaw. Screws were placed after predrilling. The devices were applied under local anesthesia from first molar to first molar in adult dentate patients at the mucogingival junction. The intermaxillary fixation was maintained for approximately 4 weeks, continued by elastics and ending with device removal at 6 weeks. The comparisons included the application time (34. 9 ± 10.1 minutes in the hybrid bar group versus 53.1 ± 5.7 minutes in the EAB group), the occurrence of wire-stick injuries (8 in EAB group only), and iatrogenic tooth root damage (5 in hybrid group only). These findings – are in keeping with the nature of the two procedures. Postoperative complications in the form of loosening of the arch bars and mucosal overgrowth were confined to the hybrid group with 5 and 7 cases respectively. Oral hygiene was more often compromised in the conventional arch bar group.
A further RCT compared the length of surgery between SMARTLock Hybrid MMF System (regular size) and conventional EABs (Bouloux 2018)[20]. The mean time for installing the devices in the 26 hybrid patients of 14 ± 8.4 minutes was statistically different to 37 ± 15.1 minutes in the 24 EAB patients. The mean time to complete the closed reduction or ORIF surgery did not differ significantly after adjusting for time-sensitive covariates such as number of fractures, fracture location and surgical method (108 ± 107,8 minutes in hybrid group versus 117 ± 57.1 minutes EAB group).
5 tooth root injuries due to screw insertion in the hybrid bar patients were the only complications seen in the study.
Two reasons were posited for the absence of a significant difference in the overall length of surgery between the groups. First, controlling and reducing the fragments by circumdental wires in a familiar and effective component of EAB application and might have been responsible for time savings in contrast to working with the less flexible embodiment of the hybrid devices. Second, the presence of support legs (lugs) and screws may have required altering the placement of intraoral soft tissue incisions from the mucogingival junction farther into the vestibule which would compromise visualization and ease of access requiring more time.
These results concurred with Chao and Hulsen (2015)[16], that hybrid arch bars can be timesavers during application but otherwise behave quite similar to conventional EABs.
Shortly thereafter, another RCT followed that examined the time for installation and removal, the effects on the gingiva, and the operator safety of the SMARTLock Hybrid MMF System compared to conventional Erich arch bars in dentate adult patients with one or more mandibular fractures (King and Christensen 2019)[21]. 90 patients were included in a parallel-group design: 47 in the hybrid arch bar group and 43 patients in the EAB group. For the hybrid group 5 self-drilling screws were inserted in each bar using a battery powered screw driver for a total of 10 screws.
Additional screws were placed if needed. The EABs were typically secured with 20 circumdental wires in total. Both devices were placed under general anesthesia and removed 6 weeks postoperatively under local anesthesia.
21 patients were lost to follow-up including 16 in the hybrid group and 5 in the EAB group.
The application time was significantly reduced in the hybrid arch bar group com-pared to the EAB group – (mean 6.9 ± 3.1 minutes versus 31.3 ± 9.3 minutes = time saving value: 24.4 minutes). The rate of glove perforations and/or tears per application as assessed by verbal questioning the operators was 0.6 ± 0.9 in the EAB group which exceeded the hybrid group (0.1 ± 0.3).
No adverse events solely attributable to hybrid arch bars, such as damaged teeth, occurred.
Grading for gingival appearance at removal of the devices did not differ. There was a slight difference in the percentages of loose hardware (number of loose screws or wires / total number), 9.4 ± 17.7 % for hybrid arch bars and 7.5 ± 10.6 % for EABs.
Removal time was significantly less with hybrid arch bars, 10.5 ± 5.1 minutes than for EABs (17.9 ± 10.7 minutes).
Glove perforations and/or tears for removal was higher with EAB (0.6 ± 0.9) than with the hybrid group (0.1 ± 0.3).

It is important to note that this comparative study is unique from all others in the accurate reporting of the number of screws and circumdental wires, thus making comparison of application times more meaningful. A two-center retrospective study reporting an in-depth cost analysis of the SMARTLock Hybrid MMF System compared to EABs was presented by Khelemsky (Khelemsky et al. 2019)[22]. Both MMF forms were used in conjunction with transoral ORIF of unilateral or bilateral mandibular fractures. Closed treatment of concomitant condylar process fractures was also included.

102 adult patients (n = 59 for the hybrid sample and n = 43 for EAB controls) were included in the study. Apart from the anatomic location of mandibular fractures and the operative ◎time no further clinical data were reported.

The authors used five MMF screws per arch, rather than seven in the study by Kendrick et al. (2016)[20] and maintained adequate rigidity reflecting the clinical practice at the two centers. The cost calculations were based on 10 bar retaining screws per case whereas the corresponding average number of circumdental wires for EABS was not specified.

The hybrid devices were more often used in unilateral fracture cases (n = 33 or 55.9 %) than bilaterally (n = 26 or 44.1 %), opposite to EABs where bilateral (n = 23 or 53.5 %) exceeded unilateral cases (n = 20 or 46.5 %). The total operative time (from incision to completion of closure), were compared by groups along with mean time savings in for the hybrid devices versus EABs for unilateral and bilateral fractures.

The average operative time was significantly shorter for the hybrid devices(136 ±2.7 minutes) than for the EAB (186.7 ± 70.7).

The operative times for bilateral fractures were longer (183.6 ± 71.1 minutes) than for unilateral fractures (133.2 minutes ± 49.57 minutes).

In the analyses within the unilateral and bilateral fracture groups the anatomic fracture location was not a significant variable. Operative times for uni- and bilateral fracture patterns resulted in significant time savings for the SMARTLock System - 37.2 ± 13.2minutes for unilateral fracture types and of 55.8±18.9 minutes for the bilateral. No explanation was put forth to explain greater time savings with the hybrid devicesin bilateral compared to unilateral fractures.

When uni- and bi- laterality of the fractures were included the time-saving effect of the hybrid arch bars still accounted for 50.8 ± 12.8 minutes.

The authors argued their findings, particularly in respect to bilateral fractures would have been missed, if the study had evaluated device application times alone. They speculated of some interdependencies of ORIF techniques and the hybrid MMF modality.The cost benefit analysis of the MMF system was based on three incremental fees for operating room utilization per minute (see paragraph on -Economics/Cost analysis).

Another randomized study (Hamid and Bede 2021)[33] compared the clinical outcomes between screw retained hybrid arch bars (SMARTLock Hybrid MMF) and conventional EABs for closed treatment of mandibular fractures.

18 patients were divided into controls (n=10) receiving EABs and an 8 patient study group where SMARTLock devices were used. The mean application time differed significantly in favor of the hybrid devices: 41.6 ± 6 minutes versus 61.6 ± 11.4 minutes for EABs. The mean time for removal was also shorter 11.1 ± 2 minutes for the hybrid MMF devices versus 14.2 ± 3 minutes for EABs. By nature screw loosening and mucosal overgrowth occurred only in the hybrid devices with percentages of 12.5% and 31.2% respectively. Glove tears were seen exclusively in EABs occurring in 70 % of cases. In the overall assessment the SMARTLock MMF System was acknowledged as a suitable alternative to EABs due to time savings in application and improved safety for surgeons.

The most recent clinical RCT comparing hybrid arch bars and EABs aimed to unveil differences in efficacy and safety in treating mandibular fractures (Sankar et al. 2023)[23]. 44 patients (age 18 – 45 years) after closed reduction and immobilization of single site minimally displaced fractures in the symphysis, body or angle were followed over a minimum of 4 weeks. Condylar process fractures were excluded. The patients had been randomized to a hybrid group (stainless steel, SMARTLock facsimile embodiment n = 21 and a conventional EAB group (n = 23).

Clinical outcomes assessed parameters were as usual: application time and stability of the arch bars, glove punctures, operator skin pricks, tooth root injuries, screw soft tissue coverage and oral hygiene.

The hybrid bars were fixed with a minimum of six 2.0 screws (lengths 6, 8 or 10 mm) per arch after an OPG (Orthopantomogram)/CBCT examination of the tooth root topography. EABs were secured with circumdental 26 G stainless steel wires and not detailed further.

Arch bar stability was assessed weekly and graded from no mobility over mild/reattachable through to perceptible requiring removal.

The efficacy of a double glove protection while applying the arch bars was checked by water-filling and compression to identify puncture holes as described by Pieper et al. (1995)[108]).

Potential tooth root injuries were screened weekly by percussion testing of the teeth. In cases of tenderness the spatial relationship between screw and root was examined by CBCT.

Tissue growth over the screw heads was indexed progressively from 0, to half (1), to more than half (2) and to complete (3) coverage for each screw. A total score was calculated using the sum of indices divided by the number or screws.

The oral hygiene status was evaluated with the Oral Hygiene Index-Simplified (OHI-S) (Greene and Vermillion 1964)[105], which tallies 3 components - debris, bleeding and gingival enlargement (compare Hassan et al. 2018)[9].

As expected the application time for the hybrid arch bars was significantly shorter than in the EAB group (55.7 ± 17.9 minutes versus 82.0 ± 12. 2 minutes).

The arch bars stability scores in both jaws were comparable at a good level and did not decrease over the follow-up period.

Outer glove and inner glove punctures were detected in 39.1% of EAB (associated with 1 operator pick) and 8.7 % in the hybrid system, respectively.

Of 252 screws used in the hybrid group, 2 screws (0.9%) in the mandibular incisor region displayed tooth root damage confirmed with CBCT imaging.

Mucosal overgrowth progressed from the 1st to the 4th post OP week with 137 (54,4 %) screws eventually completely buried.

Initially the OHI-S indices were comparable between the two groups, thereafter the oral hygiene status declined continuously in the EAB group, but maintained its level in the hybrid group.

The latest (prospective) study of hybrid devices – identical in construction with the SMARTLock MMF System – compared to conventional EABs (Burman et al. 2023)[24] matched 20 and 21 patients, respectively to the treatment arms.

The treatment indications for hybrid devices were mandibular trauma in 75 % of patients and midface fractures in 25 %. EABs were equally distributed on patients with mandibular and midface fractures. The installation of the hybrid system required general anesthesia in 95 % of the patients. The therapy was closed reduction. The hybrid devices as well as the EABs were left for 4 weeks postoperatively.

The intraoperative application time for the hybrid devices was significantly less than for EABs (mean 23.3 ± 8.1 minutes versus 86.4 ± 26.5 minutes). Up to a maximum of 18 screws (nine per arch) were used to anchor the hybrid devices.

No tooth damage was seen in patients with bone-anchored devices as assessed by vitality testing and postoperative panoramic x-rays.

Optimal restoration of preinjury occlusion was achieved in 90 % of the hybrid device patients compared to 81 % of the EAB patients.

Stability at 1 and 2 weeks postoperatively by use of a numerical score indicated that the hybrid devices had maintained its initial strength in most patients (n = 17 with the maximum score 3) whereas the EABs lost rigidity in the majority of patients (n=16 with score 2 categorized as unstable). The risk for instability was increased 3-fold in EABs.

Screw loosening or yielding of the circumdental wires, respectively occurred in six patients (30%) with hybrid devices and in 11 patients (52 %) with EABs.

Mucosal overgrowth, defined as coverage > ¾ of the screw head in the bendable flanges ('lugs') of the stainless steel hybrid devices, was found in n = 15/20 patients (75 %). By contrast Kendrick et al. (2016)[17] had observed mucosal overgrowth in 38 % of their patients. The subsequent discussion notes that conventional rather than locking screws had been used for bony fixation and led to impingement into the mucosa. Moreover, the high rate of mucosal migration over the tips and screw heads of the lugs most likely

was a result of the unfavorable placement into the mobile vestibular mucosa – which was displayed in photographs from the article.
Obviously because of mucosal overgrowth and the need for local anesthesia, the mean time to remove the hybrid devices (30 minutes) exceeded the time needed for the EABs (19 minutes).The authors stressed the superiority of the hybrid MMF devices in terms of clinical efficiency, reduced installation time and safety outweighing the disadvantage of mucosal overgrowth.
A small retrospective series compared the SMARTLock Hybrid MMF system and IMF (MMF) screws in the closed treatment of condylar and subcondylar fractures (Roeder et al. 2018)[25]. The analyzed parameters were application time, occlusal restoration, interincisal opening, TMJ dysfunction, mucosal overgrowth and overall costs.
The series included 7 patients with the hybrid modality and 5 patients with IMF screws (Synthes USA Products LLC, West Chester, PA). The series included one patient with an isolated condylar head fracture with the remaining patients with different types of condylar process fractures plus additional fractures of the mandible. The treatment for condylar process fractures was closed reduction along with ORIF for the associated non-condylar mandibular fractures in both groups. Postoperative intermaxillary fixation ranged between 3 to 5 weeks with a period of 4 weeks occurring in 80 % of cases.
The outcome in terms of occlusion, interincisal distances and TMJ complaints showed conformity within the two groups. The application time for the MMF devices was not separately recorded from the total operative time. The operative room time for patients by use of hybrid MMF only averaged to 39.6 minutes and for patients with IMF screws 43 minutes. In hybrid plus ORIF of associated fractures the mean OR time was 81.3 minutes. IMF screws plus ORIF of another fracture had a mean of 78.2 minutes. The differences in the operating room times (3 -5 minutes) were considered negligible by the authors.
In the hybrid retaining screws, mucosal overgrowth was more prevalent than in MMF screws, presumably because the SMARTLock Hybrids have low profile screw heads.
The authors report that as a result from shifting the intraoral incision sites towards the gingivobuccal sulcus, intraoral wound healing problems could not be recorded in hybrid plus ORIF procedures. Tooth root injuries were not mentioned in the report.
In general, the hybrid arch bars were felt to be advantageous in providing a ‘tension band’ function and to allow for more flexibility in the vectors of elastic loops during treatment. This was regarded relevant in the postoperative phase for complex fractures. Criticism was directed at the higher frequency of mechanical lip irritation by the hybrid system as formerly seen in conventional arch bars and for the increased costs. The authors (Roeder et al. 2018)[25] called for a prospective trial to discern the pros and cons of hybrid systems compared to IMF screws.
Two years later a randomized prospective comparison on the regular sized SMART-Lock Hybrid MMF System versus traditional IMF screws was authored (Aslam-Pervez et al. 2020)[26]. 32 Patients with singular or multiple mandibular fractures requiring intra- and postoperative intermaxillary fixation were allotted to according treatment arms: 19 patients receiving hybrid MMF and 13 patients receiving MMF screws.
Patients with foreseeable damage to unerupted permanent teeth excluded.The usual parameters were investigated: time to device application and removal, glove perforations and needle stick injuries, rate of tooth root damage, hardware failure (screw loss and loosening) and soft tissue granulation/mucosal overgrowth on screw heads. A percentage of 75 % patients underwent intermaxillary fixation only with closed reduction, 25 % had additional open reduction and internal fixation.
Self-drilling 2.0 screws (lengths 6 or 8 mm) were used in both MMF modalities. The average retaining screw number for the SMARTLock devices amounted to 9.9 (11) contrasting with 5.7 (6) MMF screws. The application time was 25.9 minutes in the hybrid group compared to 18.3 minutes with MMF screws. Glove perforations or needle-stick injuries resulting from wiring in the intermaxillary cerclages were not seen in either group. Tooth root damage not needing therapy was found in one patient of each treatment arm. Screw loosening occurred in a total of 3 cases (2 MMF cases versus 1 hybrid case). The hybrid system more often had gingival edema, erythema and soft tissue overgrowth compared to MMF screws: 8 (42 %) versus 2 (15.4%) cases, 9 (69.2 %) versus 1 (7.7 %) case and 7 (36%) against 1 (7.7 %) case, respectively. The 39% (9/23) mucosal overgrowth rate in the Hybrid devices was similar to that reported by Kendrick et al.

(2016)[20] but was well below the 60 % incidence disclosed by Nizam and Ziccardi (2013)[15]. The importance of positioning the screws in the attached gingiva next to the mucogingival junction and minimizing tissue contact and compression of the oral mucosa by employing the locking mechanism to maintain the arch bar in a" standoff" position was emphasized.
A systematic three-way comparison between the MMF techniques, Erich arch bars, MMF 4-point fixation via MMF screws and bone-supported arch bars (SMARTLock Hybrid MMF System) was made in a retrospective study with the aim to identify the best option to both limit the complications and reduce costs (Edmunds et al. 2019)[27]. The cohort included 93 adult patients with unilateral or bilateral mandibular fractures, 27 with a conventional EAB, 51 with 4-point MMF screw fixation and 15 with bone-supported arch bars. The use of concomitant ORIF was an exclusion criterion.
Clinical assessment parameters were mean times for MMF application, rates of malocclusion, malunion, nonunion, wound dehiscence, tooth damage, injury to tooth roots, infection, bar/plate fracture, screw fracture, screw loosening, and loss of screw at follow-up. Suspected tooth damages by vitality testing were clarified by orthopantomography.
The mean application times were 98.7 ± 29.6 minutes for EAB, 56.1± 15.4 minutes for hybrid arch bars and 48.8 ± 23.9 minutes for 4-point MMF screw fixation. So the operative time for 4-point fixation was 7.3 minutes shorter than for bone-supported arch bars and 49.8 minutes less than in EABs.
There was no difference in the complications such as occlusal discrepancies or screw associated problems between the three groups – hybrid arch bars 13 % (n= 2/15), 4-point fixation 10 % (n=5/51) and EABs 11% (n=3/27).
With respect to screw attributable complications 1 patient from the hybrid and one from the 4-point screw fixation group encountered a premature screw loosening while 1 patient with 4-point screw fixation had early screw loss. No instances of screw or bar/plate fractures were recorded. There were no patients with tooth root damage.
More patients treated with 4-point fixation (73 %; n = 37 /51) or EAB (63 %; n= 17/27) needed to return to the OR for hardware removal in stark contrast to 7% (1/15) in the hybrid group. This was considered to be a relevant additive factor in comparison of costs (see paragraph on - Economics/Cost analysis).
A recent RCT again compared conventional EABs (n = 31 patients), MMF screws (n = 33 patients) and the SMARTLock Hybrid System (n = 29 patients), this time in noncondylar mandible fractures treated with ORIF/miniplate osteosynthesis (Salavadi et al. 2025)[28]. The overall tendency of the results corresponds to the preceding outcome study (Edmunds et al. 2019)[27]. The times for application of the MMF devices were shorter, considerably, however – EABs 78.0 ±10.7 minutes, SMARTLock Hybrids 19.0 ±1.1 minutes, MMF screws 15.1 ±1.1 minutes – but declining in the same order. Initially occurring occlusal discrepancies at 1 week postoperatively (EAB n= 3 patients; Hybrid n =1patient, MMF screws n =7 patients), were treated with intermaxillary fixation (heavy elastics or wire ligatures) and had vanished at 4 weeks follow up. Vitality testing revealed tooth root injuries in 4 patients treated with MMF screws and 2 patients in the SMARTLock Hybrid group, none in EABs.
In contrast to MMF screws appropriate oral hygiene levels (Turesky-Gillmore-Gilman plaque index)[106] turned out difficult to maintain in EABs and Hybrids. IMF screws had an increased incidence of mucosal overgrowth 4 weeks postoperatively in MMF screws (n = 14/33 or 42 %) and Hybrids (n= 10/29 or 34 %) in comparison to EABs (n=3/31 or 9,6%)
The self-perception of oral health was monitored by the General Oral Health Assessment Index (GOHAI score – Campos et al. 2017[109]) as a validated QoL (Quality of life) - scale. The responses from patients with MMF screws topped the total scores, followed by the SMARTLock Hybrid group and EABs ranked last. The superior QoL reported by patients treated with MMF screws was attributed to the limited volume of the devices less interfering with oral functions than SMARTLock or EABs.
A conference presentation (Wilt et al. 2019)[29] addressed whether hybrid arch bars (i.e. the SMARTLock System) pose a risk to the dentition.
Postoperative axial CT slices of 50 patients receiving the hybrid MMF system were inspected for lesions caused by a total of 507 screws associated with 1340 teeth. Overall 31.5 % of these teeth showed screw contact or injuries. By way of further detail – there was damage to the periodontal ligaments (7.4%), disruption of tooth root dentin (19.8%), pulp chamber perforations (3.8%) and root fractures (0.5 %).

Maxillary teeth were affected more often than the mandibular dentition. The topographic distribution of the injuries according to tooth groups varied in an anterior to posterior direction: incisors/canines 13.7%, premolars 8 % and molars 9.8 %.
The root fractures in the mandible (n = 5) exceeded maxillary teeth (n = 1). In short, bone anchor screws for a hybrid MMF system pose an increased risk to the entire maxillary dentition as well as the anterior dentition of both jaws.
A meta-analysis undertaken by Jain et al (2021)[31]) reviewed the findings of seven separate comparative studies on bone-supported arch bars (3 on self-made hybrid EAB modifications plus 4 on the SMARTLock Hybrid System) and EABs up to 2019, all of which have been outlined previously. The study objective was to identify the better MMF modality.
The analysis included RCTs, controlled clinical trials and retrospective studies. Non-randomized trials with incomplete data were excluded. However, on closer inspection, the selection (e.g. exclusion of Chao and Hulsen (2015)[19], from the metanalysis in contrast to Khelemsky et al. 2019[22]) appears incoherent.
The included studies represented a total of 382 adult patients.
The outcome parameters extracted for analysis were duration of MMF placement, stability of the arch bars, oral hygiene, glove tears/ wire-stick punctures and tooth root damage.
The Cochrane methodology for systematic reviews of RCTs was used to analyze the data.
The resulting forest plots highlight the deficiencies and discrepancies of the individual studies in terms of missing parameters, variation of the weight across the studies, substantial heterogeneity of treatment effects, inherent bias and grading of quality of evidence.
5 [Pathak et. al 2019[8], Rani et al. 2018[19], Bouloux 2018[20], King and Christensen 2015[21], Khelemsky et al. 2019[22]] of the 7 studies compared the time required for placement of the MMF devices showing that the application of hybrid arch bars required statistically significantly less time. In fact, Khelemsky et al. (2019)[25] did not report the time required for mounting of the MMF devices but rather only the overall operative time.
Only 3 studies [Pathak et. al 2019[8], Rani et al. 2018[19], Rothe et al. 2019[7]] assessed the stability of the arch bar types, and no statistical difference was discernible.
5 studies rated oral hygiene using common indices [Hassan et al. 2018[9], King and Christensen 2015[21], Rothe et al. 2019[7]] or dichotomous data [Pathak et. al 2019[8], Rani et al. 2018[19]].
Separate analysis for the two groups found statistically significantly better indexed hygiene results and trend for better binary (Rani et al. 2018)[19] and tridented (Pathak et. al 2019)[8], results for the hybrid MMF devices.
3 studies [Pathak et. al 2019[8], Rani et al. 2018[19], Rothe et al. 2019[7]] reported glove tears / wire-stick punctures which were all seen in EABs.
5 studies [Bouloux 2018[20], King and Christensen 2015[21], Pathak et. al 2019[8], Rani et al. 2018[19], Rothe et al. 2019[7]] noted tooth root damage, which was observed - due to the treatment - was restricted to the hybrid arch bar groups in 4 studies [Bouloux 2018[20], Pathak et. al 2019[8], Rani et al. 2018[19], Rothe et al. 2019[7]].The qualitative analysis attested a high risk of bias to all 7 studies.
The quality of evidence ranged from low for oral hygiene to moderate for all the other assessment parameters.
Another confounding factor was a lack of statistical power owing to the small sample size of some studies. Nonetheless, the analysis concluded that hybrid arch bars were a better MMF option than EABs, but further, more accurate research might change this preliminary result. A 2024 systematic review by Sulistyani et al. (2024)[32] compared treatment outcomes of tooth-borne and bone-borne intermaxillary devices.
After exclusion, 13 studies remained, 4 related to the SMARTLock System (Bouloux 2018[20], Edmunds et al. 2019[27], Hamid and Bede 2021)[33], Sankar et al. 2023[23]) and 1 referring to a self-made hybrid (Pathak et. al 2019)[8]) 3 papers - all concerned with SMARTLock Systems (Edmunds et al. 2019[27], Hamid and Bede 2021)[33]) and a facsimile (Sankar et al. 2023)[23] are published more recently than those reviewed by Jain et al. (2021)[31].
The 13 publications comprised 8 studies using tooth-borne fixation: 4 applying EABs, 3 studies not specifying the arch bar type and 1 study with eyelet wiring. The type of bone-borne fixation devices varied

between MMF screws in 8 studies and hybrid or bone-supported MMF devices in 5 studies (see above). The number of patients totaled 583 with clinical outcomes evaluated according to the usual criteria and assessed for typical complications. A differentiated breakdown of the various MMF modalities in the two major treatment arms, however was not accomplished, so the SMARTLock MMF System withdrew individual appraisal. The paper's conclusion leaves ambiguity simply repeating the pros and cons of tooth-borne and bone-borne devices and left the choice of MMF device to the surgeon's experience.
The most recent systematic review and meta-analysis argues that in previous reports a maximum of 2 -3 MMF techniques only have been compared to each other (Kalluri et al 2024)[34]. Thus it raises the high claim to conduct an analysis on all existing MMF techniques.
The variety of all MMF types – traditional to modern is allocated to 5 major categories: arch bars, screw-based, wire-based, plate/splint-based and other.
Unfortunately, each of these MMF 'categories sorted by type' is heterogenous and mixes the modalities without appropriate distinctions to keep them separated for analysis based on the fundamental technique and design. As a result hybrid MMF devices are listed under the heading 'Arch Bars' as hybrid-arch bars, bone supported arch bars and modified screw-retained arch bars combined with Erich arch bars. Headless compression screws with an arch bar are designated as 'screw-based' MMF techniques besides all other kinds of IMF / MMF screws. SMARTLock™ Hybrid MMF, OmniMax™ MMF System (Zimmer Biomet) and the Matrix Wave™ Plate System (DePuy Synthes) are placed in a MMF group entitled 'Other' along with bondable buttons, wire free MMF and Mitek bone anchor skeletal MMF.
Out of 4234 articles identified in the initial literature search, 24 studies were eventually included in the systematic review with 17 studies qualifying for meta-analysis. The represented MMF techniques according to the author's terminology were MMF screws (7 /24 studies, 29 %) modified arch bars (4/24 studies, 16.6 % self-made hybrid EABs) and "Other" (in total 13/24 studies, 54 %; in detail: SMARTLock [3/24 studies, 12.5 %], OmniMax [1/24 studies, 4.1 %], Embrasure wires [4 /24 studies, 16. 2%], DIMAC wires, Leonard buttons, Vacuum formed splints, Hanger plates and Eyelet [= 5 x 1/24 studies or 5 x 4.1 %]). Conventional Erich arch bars served as the reference 'gold standard' for comparisons
in the following 3 redefined basic groups, which were composed out of the 5 aforementioned categories : "Other Arch Bars", "All Other Interventions" - including modified arch bars or non-arch bar forms of MMF such as various screw-based, wire based and plate/splint-based forms of MMF and "Other Interventions" again including non-arch bar MMF forms. The total sample size for the meta-analysis was 3109 patients with a mean of 37.6 ± 20.6 patients from each study. Despite this respectable patient number the overall data set was regarded as insufficient in quantity to allow reliable statements for a large number of the typical assessment parameters for MMF technique outcomes: overgrowth /coverage of hardware by oral mucosa, impaired wound healing, postoperative infections, intra – and postoperative stability, patient comfort or operative time for MMF removal.
A closer look on the forest plots showing the actually meta-analyzed results identifies a total of 8 studies of self-made/ modified arch bars (Rothe et al. 2018[6], Pathak et. al 2019[8], Venugopalan et al. (2020)[10]) and CHMMFS devices (SMARTLock: Chao and Hulsen (2015)[16], Bouloux 2018[20], Hamid and Bede 2021)[33], Sankar et al. 2023[23], OmniMax: Aukerman et al. 2022[36]). It appears misleading that these 8 studies – despite their hybrid bone-borne MMF type– turn up in the assessments (MMF application time, malocclusion or glove perforations/wire punctures etc.) of all 3 basic evaluation groups.
The study of Venugopalan et al. (2020)[10]), for instance detailing the clinical results on a modified Erich Arch Bar is included in two groups – "All Other nterventions" and "Other Interventions" The latter of which is surely erroneous, since that category was formerly reserved exclusively for "non-arch bar forms of MMF".
The reiteration of identical MMF techniques in the 3 basic evaluation groups also occurs with some other studies, e.g. dealing with MMF screws, embrasure wires or Leonard buttons.
In the end, the puzzling mix and remixing of identical studies into a variety of technically distinct MMF groups does not provide meaningful information or guidelines to choose an appropriate MMF type for clinical practice.

However the statistics ultimately arrive at a conclusion, where modern alternative MMF modalities yield more efficient outcomes in comparison to conventional Erich arch bars. The qualifying reservation “if appropriate” for the individual patient, was not further explained (Kalluri et al 2024)[34].

References – Text S2(eContent):

6. Rothe, T.M.; Kumar, P.; Shah, N.; Shah, R.; Kumar, A.; Das, D. Evaluation of efficacy of intermaxillary fixation screws versus modified arch bar for intermaxillary fixation. *Natl. J. Maxillofac. Surg*. **2018**, *9*, 134–139. https://doi.org/10.4103/njms.NJMS_16_18.
7. Rothe, T.M.; Kumar, P.; Shah, N.; Shah, R.; Mahajan, A.; Kumar, A. Comparative evaluation of efficacy of conventional arch bar, intermaxillary fixation screws, and modified arch bar for intermaxillary fixation. *J. Maxillofac. Oral Surg*. **2019**, *18*, 412–418. https://doi.org/10.1007/s12663-018-1110-7
8. Pathak, P.; Thomas, S.; Bhargava, D.; Beena, S. A prospective comparative clinical study on modified screw retained arch bar (SRAB) and conventional Erich's arch bar (CEAB). *Oral Maxillofac. Surg*. **2019**, *23*, 285–289. https://doi.org/10.1007/s10006-019-00766-1.
9. Hassan, S.; Farooq, S.; Kapoor, M.; Shah, A. Comparative evaluation of modified Erich’s arch bar, conventional Erich’s arch bar and intermaxillary fixation screws in maxillo-mandibular fixation: A prospective clinical study. *Int. J. Med. Res. Res. Prof*. **2018**, *4*, 41–45. https://doi.org/10.21276/ijmrp.2018.4.4.011.
10. Venugopalan, V.; Satheesh, G.; Balatandayoudham, A.; Duraimurugan, S.; Balaji, T.S. A comparative randomized prospective clinical study on modified Erich arch bar with conventional Erich arch bar for maxillomandibular fixation. *Ann. Maxillofac. Surg*. **2020**, *10*, 287–291. https://doi.org/10.4103/ams.ams_20_20.
105. Greene, J.C.; Vermillion, J.R. The simplified oral hygiene index. *J. Am. Dent. Assoc*. **1964**, *68*, 7–13. https://doi.org/10.14219/jada.archive.1964.0034.
106. Turesky, S.; Gilmore, N.D.; Glickman, I. Reduced plaque formation by the chloromethyl analogue of victamine C. *J. Periodontol*. **1970**, *41*, 41–43. https://doi.org/10.1902/jop.1970.41.41.41.18. Nizam, S.A.; Ziccardi, V.B. Use of hybrid MMF in oral and maxillofacial surgery: A retrospective review. *J. Maxillofac. Trauma (Ed. Minerva Medica)* **2014**, *3*, 1–8.19. Chao, A.H.; Hulsen, J. Bone-supported arch bars are associated with comparable outcomes to Erich arch bars in the treatment of mandibular fractures with intermaxillary fixation. *J. Oral Maxillofac. Surg*. **2015**, *73*, 306–313. https://doi.org/10.1016/j.joms.2014.08.025.
16. Chao, A.H.; Hulsen, J. Bone-supported arch bars are associated with comparable outcomes to Erich arch bars in the treatment of mandibular fractures with intermaxillary fixation. *J. Oral Maxillofac. Surg*. **2015**, *73*, 306–313. https://doi.org/10.1016/j.joms.2014.08.025.
17. Kendrick, D.E.; Park, C.M.; Fa, J.M.; Barber, J.S.; Indresano, A.T. Stryker SMARTLock Hybrid Maxillomandibular Fixation System: Clinical application, complications, and radiographic findings. *Plast. Reconstr. Surg*. **2016**, *137*, 142e–150e. https://doi.org/10.1097/PRS.0000000000001920.
18. Kendrick, D.E.; Park, C.M. Reply: Stryker SMARTLock Hybrid Maxillomandibular Fixation System: Clinical application, complications, and radiographic findings. *Plast. Reconstr. Surg*. **2016**, *138*, 949e–950e. https://doi.org/10.1097/PRS.0000000000002730.
19. Rani, E.B.; Reddy, S.; Amarnath, K.; Suresh Kumar, M.; Visalakhshi, G. Bone supported arch bar versus Erich arch bar for intermaxillary fixation: A comparative clinical study in maxillofacial fractures. *Int. J. Curr. Res.* **2018**, *10*, 69848–69850.
20. Bouloux, G.F. Publication: Does the use of hybrid arch bars for the treatment of mandibular fractures reduce the length of surgery? *J. Oral Maxillofac. Surg*. **2018**, *76*, 2592–2597. https://doi.org/10.1016/j.joms.2018.06.172.
21. King, B.J.; Christensen, B.J. Hybrid arch bars reduce placement time and glove perforations compared with Erich arch bars during the application of intermaxillary fixation: A randomized controlled trial. *J. Oral Maxillofac. Surg*. **2019**, *77*, e1–e1228. https://doi.org/10.1016/j.joms.2019.01.030.
22. Khelemsky, R.; Powers, D.; Greenberg, S.; Suresh, V.; Silver, E.J.; Turner, M. The hybrid arch bar is a cost-beneficial alternative in the open treatment of mandibular fractures. *Craniomaxillofac. Trauma Reconstr*. **2019**, *12*, 128–133. https://doi.org/10.1055/s-0038-1639351.
23. Sankar, H.; Rai, S.; Jolly, S.S.; Rattan, V. Comparison of efficacy and safety of hybrid arch bar with Erich

arch bar in the management of mandibular fractures: A randomized clinical trial. *Craniomaxillofac. Trauma Reconstr*. **2023**, *16*, 94–101. https://doi.org/10.1177/19433875221080019.
24. Burman, S.; Rao, S.; Ankush, A.; Uppal, N. Comparison of hybrid arch bar versus conventional arch bar for temporary maxillomandibular fixation during treatment of jaw fractures: A prospective comparative study. *J. Korean Assoc. Oral Maxillofac. Surg*. **2023**, *49*, 332–338. https://doi.org/10.5125/jkaoms.2023.49.6.332.
25. Roeder, R.A.; Guo, L.; Lim, A.A. Is the SMARTLock Hybrid Maxillomandibular Fixation System comparable to intermaxillary fixation screws in closed reduction of condylar fractures? *Ann. Plast. Surg*. **2018**, *81* (6S Suppl 1), S35–S38. https://doi.org/10.1097/sap.0000000000001497.
26. Aslam-Pervez, N.; Caccamese, J.F.; Warburton, G., Jr. A randomized prospective comparison of maxillomandibular fixation (MMF) techniques: "SMARTLock" hybrid MMF versus MMF screws. *Oral Surg. Oral Med. Oral Pathol. Oral Radiol*. **2020**, *130*, 640–644. https://doi.org/10.1016/j.oooo.2020.07.015.
27. Edmunds, M.C.; McKnight, T.A.; Runyan, C.M.; Downs, B.W.; Wallin, J.L. A clinical comparison and economic evaluation of Erich arch bars, 4-point fixation, and bone-supported arch bars for maxillomandibular fixation. *JAMA Otolaryngol. Head Neck Surg*. **2019**, *145*, 536–541. https://doi.org/10.1001/jamaoto.2019.0183.
28. Salavadi, R.K.; Sowmya, J.; Mani Kumari, B.; Kamath, K.P.; Anand, P.S.; Kumar, N.M.R.; Jadhav, P. Comparison of the efficacy of Erich arch bars, IMF screws and SMART Lock Hybrid arch bars in the management of mandibular fractures—A Randomized clinical study. *J. Stomatol. Oral Maxillofac. Surg*. **2025**, 102217. https://doi.org/10.1016/j.jormas.2025.102217.
29 Wilt, D.; Kim, C.; StJohn, D. Do hybrid arch bars pose a risk to the dentition ? In Proceedings of the American College of Oral and Maxillofacial Surgeons—40th Annual Scientififc Conference and Exhibition, Santa Fe, NM, USA, 7–9 April 2019.
30. Carlson, A.R.; Shammas, R.L.; Allori, A.C.; Powers, D.B. A technique for reduction of edentulous fractures using dentures and SMARTLock Hybrid Fixation System. *Plast. Reconstr. Surg. Glob. Open* **2017**, *5*, e1473. https://doi.org/10.1097/gox.0000000000001473.
31. Jain, A.; Taneja, S.; Rai, A. What is a better modality of maxillomandibular fixation: Bone-supported arch bars or Erich arch bars? A systematic review and meta-analysis. *Br. J. Oral Maxillofac. Surg*. **2021**, *59*, 858–866. https://doi.org/10.1016/j.bjoms.2021.01.004.
32. Sulistyani, L.D.; Ariawan, D.; Julia, V.; Latief, M.A.; Utomo, Y.A.; Heriasti, M.D.; Santiago, S.; Utami, D.D.; Ristiawan, I. Treatment outcome comparison between tooth borne vs bone borne intermaxillary fixation devices-a systematic review. *J. Int. Dent. Med. Res*. **2024**, *17*, 435–444.
33. Hamid, S.T.; Bede, S.Y. The use of screw retained hybrid arch bar for maxillomandibular fixation in the treatment of mandibular fractures: A comparative study. *Ann. Maxillofac. Surg*. **2021**, *11*, 247–252. https://doi.org/10.4103/ams.ams_35_21.
34. Kalluri, M.H.; Edalatpour, A.; Thadikonda, K.M.; Blum, J.D.; Garland, C.B.; Cho, D.Y. Patient outcomes and complications following various maxillomandibular fixation techniques: A systematic review and meta-analysis. *J. Plast. Reconstr. Aesthetic Surg*. **2024**, *92*, 151–176. https://doi.org/10.1016/j.bjps.2024.02.075.
36. Aukerman, W.; Dodson, B.; Simunich, T.; Shayesteh, K. Comparison of Biomet Omnimax(©) versus traditional arch bar placement in trauma patients with facial fractures. *Am. Surg*. **2022**, *88*, 523–524. https://doi.org/10.1177/0003134820960018.
108. Pieper, S.P.; Schimmele, S.R.; Johnson, J.A.; Harper, J.L. A prospective study of the efficacy of various gloving techniques in the application of Erich arch bars. *J. Oral Maxillofac. Surg*. **1995**, *53*, 1174–1176; discussion 1177. https://doi.org/10.1016/0278-2391(95)90628-2.
109. Campos, J.; Zucoloto, M.L.; Bonafé, F.S.S.; Maroco, J. General Oral Health Assessment Index: A new evaluation proposal. *Gerodontology* **2017**, *34*, 334–342. https://doi.org/10.1111/ger.12270.

• *Text S3*

- **SMARTLock Hybrid MMF System 3.5.4 & 3.5.5**

3.5.4 SMARTLock Hybrid MMF System – Economics / Cost Analyses

Interestingly enough almost all the previous clinical studies went into a thorough analysis of the economic and financial issues of the SMARTLock devices, since commercial hybrid MMF devices were exceptionally higher-priced products compared to earlier MMF options. The cost calculations per case for an MMF modality included equations between the differentials in product prices for the hardware, device application time, total operating time, operating room costs, device removal costs, personnel expenses for nursing, OR technician, surgical staff and anesthesia fees. In general, the time for MMF device installation was regarded as most essential in the financial computations. It must be un- derscored, that the time required depends on the lengths of the used bars, the number of anchoring screws or circumdental wires fixations and the type of insertion tools.

Secondary procedures, such as the need for touch-ups or repairs during follow-up and the charges for MMF / arch bar removal in the office or in the OR under general anesthesia can further increase costs (Edmunds et al. 2019)[30].

Cost breakdowns in the literature were inconsistent and in part even contradictory – such that the SMARTLock System was determined to be the most expensive (e.g. Nizam and Ziccardi (2013)[18], (Roeder et al. 2018)[28]), cost-neutral (e.g. Chao and Hulsen (2015)[19], Kendrick et al. (2016)[20]) as well as the most cost-effective MMF solution (King and Christensen 2015[24], Khelemsky et al. (2019)[25], Edmunds et al. 2019[30]). In light of these disparities, which could be a consequence of principal differences and changes in the health care compensation models, e.g. fixed budgets or capped refinancing rates, and even currency variations (Sankar et al. 2023)[26], it is likely inappropriate to characterize the financial aspects of MMF devices here. These financial aspects are not transferable internationally and there are distinct differences between departments and institutions.

3.5.5 SMARTLock Hybrid MMF System – Extended Range of Applications

SMARTLock Hybrid MMF System can be used in the management of facial fractures in edentulous patients. The use of preexisting dentures or newly fabricated Gunning splints in conjunction with arch bars or MMF screws is common practice.

A case report details how to use the small size SMARTLock Hybrid arch bars for this purpose (Carlson et al. 2017)[30]. The patient's dentures were furnished with the screw retained arch bars. The smallest length screws included within the SMARTLock System (6 mm) were recommended for the fixation in the dentures pink resin base. Gliding holes were drilled then through the flanges of the fused arch bar/denture splints in locations anatomically appropriate for fixation in accessible maxillary or mandibular alveolar ridges with 10 mm screws. Intermaxillary stainless steel ligatures were wired into the connector hooks (‚cleats') of the opposing bars to bring the upper and lower arches into occlusion.

Post treatment, the splints can be easily removed, and disassembled from the bar to accomplish repair of the screw damage sites with selfcuring dental resin.

This procedure can be certainly considered as simple, readily available and rapid but it does not represent an entirely novel technique as asserted by the authors. To place conventional screws, MMF screws or hanger plates directly into the edentulous alveolar processes to provide anchor points for intermaxillary fixation above or below the margins of partial or full dentures

and thereby completely preserving their integrity was reported by Win (Win et al. 1991)[41].

Another modification proposed to fixing the dentures with MMF screws into the alveolar processes and using the protruding screw heads for intermaxillary fixation (Newaskar et al. 2013[42], Chaudhary et al. 2014[43]).

References – Text S3 (eContent):

15. Nizam, S.A.; Ziccardi, V.B. Use of hybrid MMF in oral and maxillofacial surgery: A retrospective review. *J. Maxillofac. Trauma (Ed. Minerva Medica)* **2014**, *3*, 1–8.19. Chao, A.H.; Hulsen, J. Bone-supported arch bars are associated with comparable outcomes to Erich arch bars in the treatment of mandibular fractures with intermaxillary fixation. *J. Oral Maxillofac. Surg*. **2015**, *73*, 306–313. https://doi.org/10.1016/j.joms.2014.08.025.
16. Chao, A.H.; Hulsen, J. Bone-supported arch bars are associated with comparable outcomes to Erich arch bars in the treatment of mandibular fractures with intermaxillary fixation. *J. Oral Maxillofac. Surg*. **2015**, *73*, 306–313. https://doi.org/10.1016/j.joms.2014.08.025.
17. Kendrick, D.E.; Park, C.M.; Fa, J.M.; Barber, J.S.; Indresano, A.T. Stryker SMARTLock Hybrid Maxillomandibular Fixation System: Clinical application, complications, and radiographic findings. *Plast. Reconstr. Surg*. **2016**, *137*, 142e–150e. https://doi.org/10.1097/PRS.0000000000001920.
21. King, B.J.; Christensen, B.J. Hybrid arch bars reduce placement time and glove perforations compared with Erich arch bars during the application of intermaxillary fixation: A randomized controlled trial. *J. Oral Maxillofac. Surg*. **2019**, *77*, e1–e1228. https://doi.org/10.1016/j.joms.2019.01.030.
22. Khelemsky, R.; Powers, D.; Greenberg, S.; Suresh, V.; Silver, E.J.; Turner, M. The hybrid arch bar is a cost-beneficial alternative in the open treatment of mandibular fractures. *Craniomaxillofac. Trauma Reconstr*. **2019**, *12*, 128–133. https://doi.org/10.1055/s-0038-1639351.
23. Sankar, H.; Rai, S.; Jolly, S.S.; Rattan, V. Comparison of efficacy and safety of hybrid arch bar with Erich arch bar in the management of mandibular fractures: A randomized clinical trial. *Craniomaxillofac. Trauma Reconstr*. **2023**, *16*, 94–101. https://doi.org/10.1177/19433875221080019.
25. Roeder, R.A.; Guo, L.; Lim, A.A. Is the SMARTLock Hybrid Maxillomandibular Fixation System comparable to intermaxillary fixation screws in closed reduction of condylar fractures? *Ann. Plast. Surg*. **2018**, *81* (6S Suppl 1), S35–S38. https://doi.org/10.1097/sap.0000000000001497.
27. Edmunds, M.C.; McKnight, T.A.; Runyan, C.M.; Downs, B.W.; Wallin, J.L. A clinical comparison and economic evaluation of Erich arch bars, 4-point fixation, and bone-supported arch bars for maxillomandibular fixation. *JAMA Otolaryngol. Head Neck Surg*. **2019**, *145*, 536–541. https://doi.org/10.1001/jamaoto.2019.0183.
30. Carlson, A.R.; Shammas, R.L.; Allori, A.C.; Powers, D.B. A technique for reduction of edentulous fractures using dentures and SMARTLock Hybrid Fixation System. *Plast. Reconstr. Surg. Glob. Open* **2017**, *5*, e1473. https://doi.org/10.1097/gox.0000000000001473.
110. Win, K.K.; Handa, Y.; Ichihara, H.; Tatematsu, N.; Fujitsuka, H.; Ohkubo, T. Intermaxillary fixation using screws. Report of a technique. *Int. J. Oral Maxillofac. Surg*. **1991**, *20*, 283–284.
111. Newaskar, V.; Agrawal, D.; Idrees, F.; Patel, P. Simple way of fixing a Gunning-type splint to the bone using intermaxillary fixation screws: Technical note. *Br. J. Oral Maxillofac. Surg*. **2013**, *51*, e59–e60. https://doi.org/10.1016/j.bjoms.2012.04.075.
112. Chaudhary, Z.; Sharma, R.; Krishnan, S. Maxillo Mandibular Fixation in Edentulous Scenarios: Combined MMF Screws and Gunning Splints. *J. Maxillofac. Oral Surg*. **2014**, *13*, 213–214. https://doi.org/10.1007/s12663-013-0481-z.

- ***Text S4***

- **Standoff function and overall stability of CHMMFS**

The ability for engagement at variable angles and angular stability are valuable fea tures for mechanical interlocking between a screw and plate. The Stryker SMARTLock brochure refers to pioneering work of D. Wolter (US Patent No. 4794918[113], Wolter et al 1999[114]) on internal fixator systems for osteosynthesis, so that a screw insertion and stable ixation with a slight inclination to the perpendicular axis of the hybrid MMF plate is admissible. Another indication is the tapered design of the conical locking heads with incremental sizes of the truncated threads. The locking head of the OmniMax locking screws has a cylinder shape along with truncated V-threads of identical size in parallel. The vertical height as well as the diameter of this cylindrical locking head exceeds all other locking screws of commercial MMF hybrids (Fig. 15).

The OmniMax locking screws are best inserted at right angles to the slot or plate embodiment thereby driving the plate safely into the annular retention groove just below the screw head and achieve optimal seating and angular stability for the soft-tissue stand-off.

Once seated perpendicularly, the standoff of the bar can be adjusted upwards or downwards with turns of the screw into or out of the bone.

However a stripping of the bone threads compromising the grip of the screw shaft in the bone can occur. The OmniMax slots are beveled, so that a screw insertion at angles up to 10 degrees is possible. Excess tilting and irregular (non-collinear) alignment between the screw's locking head and the beveled slot, however, will cause friction, jamming and cold welding of the colliding fringes before the terminal standoff position and maximum depth into the plate is reached.

The position of the plate above the soft tissue surface also depends on the degree of screw angulation and twisting. A lengthways screw deviation may be less relevant, since the slots do not form a closed ring around the threads of the locking head like a plate hole. Interestingly D. Wolter (1999)[55] intentionally relied on friction grip effects to realize the locking mechanism. Due to differing degrees of hardness – harder titanium self-tapping screws ground into a softer Titanium plate to create permanent bonding.

The L1 MMF locking screw has a pronounced conical locking head with V-shaped threads permitting an insertion at variable angles. In addition the slider plate with a beveled screw receiving hole is separate from the MMF bar/rack and provides three dimensional moving options (Fig. 12). Thus the slider plate position can flexibly adapt in space between the conical locking head and the coupling site at the bar /rack to counterbalance angular deviations and allow for the desired standoff. Given these multiple interfaces for offset, the L1 MMF locking screw will consistently lift the slider plate until it is flush beneath the flat screw head.

The Matrix Wave locking screw features a conical locking head with sharp V-shaped thread circles of equal size on top and on bottom and larger threads bulging at the center. Distinct from the other three CHMMFSs the screw receiving holes of the MWP begin with a bevel (countersink) at the top side and continue into two tapered threads. The reciprocal interplay between the conical locking head and the hole threads allows for fixed angles up to 15 degrees.

With any larger deviation from the vertical axis, the screw insertion will come to a standstill because the threads of the conical locking head and the plate hole threads no longer fit together and become crossed. With the plate leading to contact the mucosa or the standoff height getting unacceptably low.

Overtightening of the screws causes the MWP to rotate. So in the final phase of insertion each screw must be gently secured to prevent mechanical damage to the plate, locking hole, screw or bone.

References – Text S4(eContent):

113. Wolter, D. Bone Plate Arrangement. US Patent No. 4,794,918, 3 January 1989.

114. Wolter, D.; Schümann, U.; Seide, K. Universeller Titanfixateur interne.Entwicklungsgeschichte, Prinzip,Mechanik, Implantatgestaltung und operativer Einsatz. [The universal titanium internal fixator. Development, mechanics, implant design, and surgical application]. *Trauma Berufskrankheit*. **1999**, *1*, 307–319.

https://doi.org/10.1007/s100390050055.

• *Text S5*

• Technical features of the CHMMFS screws

The threaded shafts of MMF screws from the hybrid MMF device league serve for bone insertion. They all have single start threads which are oriented in right handed direction. The thread design is fine-pitched, straight along the cylindrical shafts and tapered towards the screw tips as in wood screws.The cross sectional shape of the threads of all four hybrid MMF screws are triangular (Fig. 15). The V-shaped bone threads of the SMARTLock screw are truncated and thereby have a trapezoidal profile which offers high strength. The L1 MMF locking screw as well as the Matrix Wave screw have sharp V-threads while the threadform of the OmniMax screw shaft is scalene. The latter thread profile is asymmetric having one square face opposed to a slanted flank, similar to a saw tooth.

The outer diameter or major thread diameter of the SMARTLock, OmniMax and L1 MMF locking MMF screw shafts is 2.0 mm while the Matrix Wave screw is 1.85 mm.

The SMARTLock and L1 MMF locking screws come in two overall lengths of 6 and 8 mm. The manufacturers specifications on the lengths of the OmniMax and the Matrix Wave locking screws do not contain the height of the conical locking heads and screw heads. The OmniMax screws have bone insertion shafts in three lengths – 7.0 mm, 9.0 mm and 11.0 mm. The Matrix Wave locking screws are available with a shaft length of 6 mm or 8 mm.

All four CHMMFSs are equipped with press-fit, friction-fit or self-retaining screw driver blades. secure engagement between the recesses in the screw heads and the blade tip is established by firmly compressing the interfacing components. For the SMARTLock System a blade with a holding sleeve is also available.The blades can be combined with conventional or ratcheting handles. In practical use sleeve-cuffed blades - although the sleeves with release rings are inherently larger than blades alone - are associated with a lower frequency of screw disconnections.This must appear especially advantageous in difficult to access areas of the oral cavity.

References – Text S5(eContent):

- None

- ***Text S6***

- **CHMMFS – Segmentation, Tension Bending, Minimum Screw Count for Fixation**

The design (e.g. single bevel versus plural threads) and mechanical (e.g. shear strength) properties of the screw receiving hole in the hybrid MMF system require consideration in relation to matching of the components, their precision fit and the stability of the standoff.

The assembly of several (≥ 2) MMF locking screws and hybrid plate/bar segments produces a compound or monobloc construction, – oftentimes labeled as 'tension band' – wherein all screws effectively behave in unison.

The MWPs require bridal wires between neighboring screws to provide a transverse monobloc or indeed a tension band function if an intermediate fracture line is crossed to prevent splay.

The number of locking screws for osseous anchorage fundamentally determines the stability of a hybrid MMF framework. The number of locking screws which has actually been used, is generally referred to as for installation of the hybrid MMF bars/devices per arch or per case. For SMARTLock bar applications the number varies between 5 to 7 per arch or 10 to 12 to 14 per case (Table S2).

A minimum of 3 screws per bar is recommended for the fixation of the OmniMax System. No more than two consecutive slots of a bar should be left empty. For the use of a bar at full length (6 segments with 12 slots) the later ruling (OmniMax Application Guide - Zimmer Biomet) would require 4 screws per bar at least.

A minimum of 5 fixation points per bar of the L1 MMF System via coaction of slider plates and locking screws is endorsed (L1 MMF Technique Guide - KLS Martin). This applies for the 7-hole and the 9-hole length. The handling at shorter lengths is not disclosed.

Matrix Wave mono- or multi- segment partitions require a locking screw insertion in each locking hole. To leave out a screw at a fastening locus would weaken the ensemble.

It is clear that reducing the screw number shortens the placement time for a hybrid MMF system. The reduction in screw number must not jeopardize stress-shielding of the entire assembly or drop its rigidity below the loading thresholds of the jaw opening muscles (Kendrick et al. 2016[17], Aslam-Pervez et al. 2020[26]).

However the knowledge of physiological jaw opening forces in healthy adults is very limited. Technical measurement tools and assessment protocols vary greatly in the literature, consequently the data are far from reliable (Greenbaum et al. 2022)[115]. There is a wide 1289
range of variation, for instance in a population of 149 participants aged between 20 – 60 years men had greater opening forces with a median of 79.0 N, an IQR (Interquartile Range) of 63.86 N and a maximum value of 166, 61 in comparison to women with a median of 41.16 N, an IQR of 30.44 N and a maximum value of 157.77 N (Brunton et al. 2017)[116]. This lack of solid physiological references may be one of the reasons that the limits of stability and resistance of hybrid MMF systems to jaw opening forces have not yet been simulated in vitro or in bench studies

References – Text S6 (eContent):

17. Kendrick, D.E.; Park, C.M.; Fa, J.M.; Barber, J.S.; Indresano, A.T. Stryker SMARTLock Hybrid Maxillomandibular Fixation System: Clinical application, complications, and radiographic findings. *Plast. Reconstr. Surg*. **2016**, *137*, 142e–150e. https://doi.org/10.1097/PRS.0000000000001920.

26. Aslam-Pervez, N.; Caccamese, J.F.; Warburton, G., Jr. A randomized prospective comparison of maxillomandibular fixation (MMF) techniques: "SMARTLock" hybrid MMF versus MMF screws. *Oral Surg. Oral Med. Oral Pathol. Oral Radiol*. **2020**, *130*, 640–644. https://doi.org/10.1016/j.oooo.2020.07.015.

115. Greenbaum, T.; Pitance, L.; Kedem, R.; Emodi-Perlman, A. The mouth-opening muscular performance in adults with and without temporomandibular disorders: A systematic review. *J Oral Rehabil*. **2022**, *49*, 476–494.https://doi.org/10.1111/joor.13303.

116. Brunton, P.A.; Loch, C.; Waddell, J.N.; Bodansky, H.J.; Hall, R.; Gray, A. Estimation of jaw-opening forces in adults. *Orthod Craniofac Res*.**2018**, *21*, 57–62. https://doi.org/10.1111/ocr.12215.

• *Text S7*

• Interradicular Screw Insertion Sites–Details.

The intraoral transmucosal (non-incision) insertion sites for bone screws have seen intensive research in vitro and clinically by means of multiplanar and 3D imaging techniques (CBCT, CT). Orthodontic miniscrews have similar diameters between 1.2 mm to 2 mm like specialized MMF screws (core diameters 1.35 mm – 1.6 mm and thread diameters up to 2 mm) or like the intrabony shaft of the fixation screws for hybrid MMF devices (core diameters 1.5 mm – 1.8 mm and thread diameters between 1.85 mm – 2 mm). So there is a valid reasoning to transfer the findings for transalveolar screw application in the adult dentition from the buccal / labial side. The ideal low-risk placement of locking screws with hybrid MMF arch bars or devices can be compromised by the fracture pattern (fragmentation and fracture line within or in direct proximity to the preferred insertion sites). The quantity and quality of the interradicular spaces for the placement of orthodontic miniscrews has been investigated in great detail. In this sense the values of the mesiodistal and bucco-/ labio-lingual dimensions were accurately collected and listed in extensive spreadsheets for the anterior (Alsamak et al. 2013[117], Shalchi et al. 2021[118]) and the posterior buccal interradicular spaces of the maxillae and mandible at incremental steps from the alveolar crest or cemento-enamel junction towards the apex (Haddad and Saddeh 2019[61], Poggio et al. 2006[67]) according to age, gender (Fayed et al. 2010)[119], ethnicity, dentoalveolar arch form and facial growwth pattern (Limeres Posse et al. 2022[120], Hasani et al. 2023[121]).
Furthermore the interradicular buccal cortical bone thickness, the density of the compact bone at the labial/buccal and lingual /palatal aspect, respectively and the density of the cancellous bone inside the cortical plates, i.e. the intercortical bone density, in respect of the demographic variables were recorded (e.g. Park and Cho 2009 [70], Li et al. 2014[122], Nucera et al. 2017[123], 2019[124]) The extensive data from the studies referenced above on suitable placement of orthodontic miniscrews can be condensed into a few recommended "safe"or "rather low risk" zones.

References – Text S7(eContent):

117. Alsamak, S.; Psomiadis, S.; Gkantidis, N. Positional guidelines for orthodontic mini-implant placement in the anterior alveolar region: A systematic review. *Int. J. Oral. Maxillofac. Implants* **2013**, *28*, 470–479. https://doi.org/10.11607/jomi.2659.
118. Shalchi, M.; Kajan, Z.D.; Shabani, M.; Khosravifard, N.; Khabbaz, S.; Khaksari, F. Cone-Beam Computed Tomographic Assessment of Bone Thickness in the Mandibular Anterior Region for Application of Orthodontic Mini-Screws. *Turk. J. Orthod.* **2021**, *34*, 102–108. https://doi.org/10.5152/TurkJOrthod.2021.20070.
61. Haddad, R.; Saadeh, M. Distance to alveolar crestal bone: A critical factor in the success of orthodontic mini-implants. *Prog. Orthod.* **2019**, *20*, 19. https://doi.org/10.1186/s40510-019-0273-1.
67. Poggio, P.M.; Incorvati, C.; Velo, S.; Carano, A. "Safe zones": A guide for miniscrew positioning in the maxillary and mandibular arch. *Angle Orthod.* **2006**, *76*, 191–197. https://doi.org/10.1043/0003-3219(2006)076[0191:Szagfm]2.0.Co;2.
119. Fayed, M.M.; Pazera, P.; Katsaros, C. Optimal sites for orthodontic mini-implant placement assessed by cone beam computed tomography. *Angle Orthod.* **2010**, *80*, 939–951. https://doi.org/10.2319/121009-709.1.
120. Limeres Posse, J.; Abeleira Pazos, M.T.; Fernandez Casado, M.; Outumuro Rial, M.; Diz Dios, P.; Diniz-Freitas, M. Safe zones of the maxillary alveolar bone in Down syndrome for orthodontic miniscrew placement assessed with cone-beam computed tomography. *Sci. Rep.* **2019**, *9*, 12996. https://doi.org/10.1038/s41598-019-49345-0.
121. Hasani, M.; Afzoon, S.; Karandish, M.; Parastar, M. Three-dimensional evaluation of the cortical and cancellous bone density and thickness for miniscrew insertion: a CBCT study of interradicular area of adults with different facial growth pattern. *BMC Oral Health.* **2023**, *23*, 753. https://doi.org/10.1186/s12903-023-03440-x.
70. Park, J.; Cho, H.J. Three-dimensional evaluation of interradicular spaces and cortical bone thickness for the placement and initial stability of microimplants in adults. *Am J Orthod Dentofacial Orthop.* **2009**;136,314 e1-12; discussion 314-315. https://doi.org/10.1016/j.ajodo.2009.01.023.
122. Li, H.; Zhang, H.; Smales, R.J.; Zhang, Y.; Ni, Y.; Ma, J.; Wang, L. Effect of 3 vertical facial patterns on alveolar bone quality at selected miniscrew implant sites. *Implant Dent.* **2014**, 23, 92-97. https://doi.org/10.1097/ID.0000000000000036.
123. Nucera, R.; Lo Giudice, A.; Bellocchio, A.M.; Spinuzza, P.; Caprioglio, A.; Perillo, L.; Matarese, G.; Cordasco, G. Bone and cortical bone thickness of mandibular buccal shelf for mini-screw insertion in adults. *Angle Orthod.* **2017**, *87*, 745–751. https://doi.org/10.2319/011117-34.1
124. Nucera, R.; Bellocchio, A.M.; Oteri, G.; Farah, A.J.; Rosalia, L.; Giancarlo, C.; Portelli, M. Bone and cortical bone characteristics of mandibular retromolar trigone and anterior ramus region for miniscrew insertion in adults. *Am. J. Orthod. Dentofac. Orthop.* **2019**, *155*, 330–338. https://doi.org/10.1016/j.ajodo.2018.04.025.
